# DICER1 Syndrome: *DICER1* Mutations in Rare Cancers

**DOI:** 10.3390/cancers10050143

**Published:** 2018-05-15

**Authors:** Jake C. Robertson, Cheryl L. Jorcyk, Julia Thom Oxford

**Affiliations:** 1 Department of Biological Sciences, Boise State University, Boise, ID 83725-1515, USA; jakerobertson719@u.boisestate.edu (J.C.R.); cjorcyk@boisestate.edu (C.L.J.); 2 Biomolecular Research Center, Boise State University, Boise, ID 83725-1511, USA

**Keywords:** DICER1 syndrome, *DICER1* germline mutations, miRNA, rare genetic disorder, cancer

## Abstract

DICER1 syndrome is a rare genetic disorder that predisposes individuals to multiple cancer types. Through mutations of the gene encoding the endoribonuclease, Dicer, DICER1 syndrome disrupts the biogenesis and processing of miRNAs with subsequent disruption in control of gene expression. Since the first description of DICER1 syndrome, case reports have documented novel germline mutations of the *DICER1* gene in patients with cancers as well as second site mutations that alter the function of the Dicer protein expressed. Here, we present a review of mutations in the *DICER1* gene, the respective protein sequence changes, and clinical manifestations of DICER1 syndrome. Directions for future research are discussed.

## 1. Introduction

DICER1 syndrome, or pleuropulmonary blastoma familial tumor susceptibility syndrome (ORPHA: 284343; OMIM: 601200), is a rare genetic disorder predisposing individual to the development of tumors, both benign and malignant [1,2,3]. Recently, mutations documented in endocrine tumors (thyroid, parathyroid, pituitary, pineal gland, endocrine pancreas, paragangliomas, medullary, adrenocortical, ovarian, and testicular tumors) have been reviewed [4]. One copy of the altered gene is sufficient to cause an increased risk of developing tumors; however, many individuals who carry a mutation in the *DICER1* gene do not develop abnormal growths. Patients may acquire a second mutation during tumorigenesis that has the potential to affect the catalytic activity of the enzyme. The prevalence of DICER1 syndrome is currently unknown and the full spectrum of clinical manifestation may not yet be fully defined [2,5,6]. However, documented cases of DICER1 syndrome have been linked to pleuropulmonary blastomas, cystic nephroma, rhabdomyosarcoma, multinodular goiter, thyroid cancer, ovarian Sertoli–Leydig cell tumors, and other neoplasias [7,8,9]. DICER1 syndrome may also include neuroblastoma [10].

**Figure 1:**
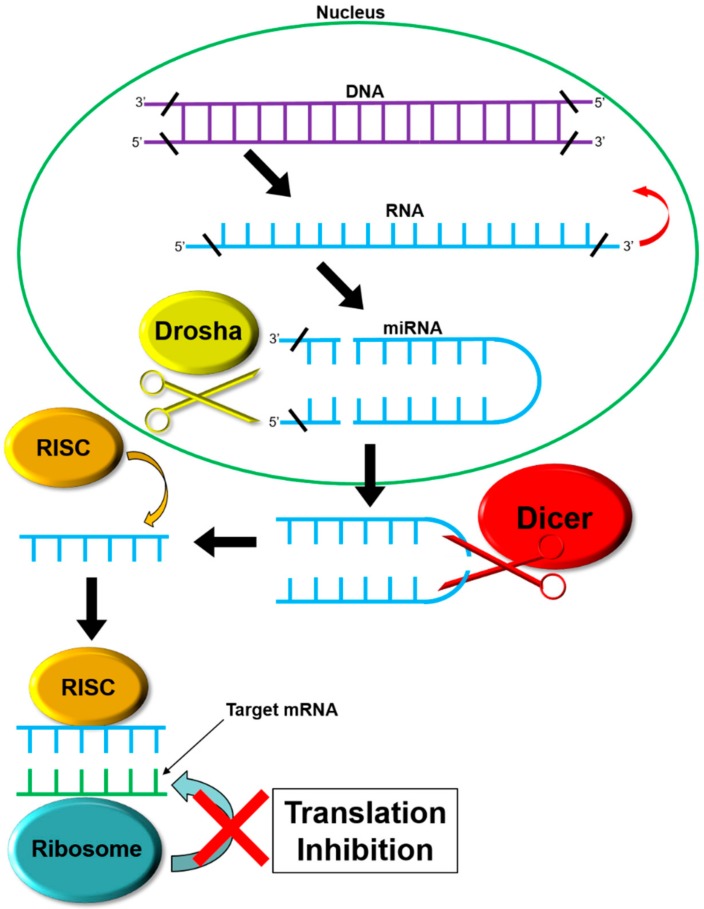
miRNA Production Pathway. DNA sequences are transcribed into RNA sequences that form a ‘hairpin’ structure of precursor miRNA. Drosha, a nucleic endoribonuclease, cleaves the hairpin from the primary RNA strand. Transported out of the nucleus by exportin 5, they are further processed by Dicer. After processing by Dicer and its accessory proteins, the hairpin structure of the precursor miRNA is degraded, leaving a single, linear piece of miRNA (the opposing piece is degraded by intracellular processes). This single piece is then bound by the RNA-induced silencing complex RISC. The RISC-miRNA complex binds to target mRNA strands, inhibiting translation by the ribosome.

The *DICER1* gene, located on chromosome 14, position q32.13, encodes the endoribonuclease Dicer protein of the ribonuclease III family. Discovered in 2001 by Bernstein, the Dicer endoribonuclease plays a role in protein translational control [11]. MicroRNAs (miRNAs) are created by the Dicer endoribonuclease protein [12,13,14]. Dicer is a component of the RNA-induced silencing complex (RISC) loading complex (RLC), which is composed of Dicer, Argonaute-2 (AGO2), and trans-activation-responsive RNA binding protein 2 (TARBP2). Dicer carries out its function downstream of DROSHA, a nuclear homolog of Dicer. miRNAs are produced from hairpin-folded pre-miRNAs that are approximately 60 nucleotides long. The pre-miRNAs are exported to the cytoplasm by exportin 5, where they are then processed by Dicer into the ~22 nt double stranded RNAs (dsRNAs). dsRNAs are loaded into the Argonaut family member protein by the RLC. The resulting RISC asymmetrically processes the ~22 nt RNA at specific 3′-overhang and 5′-phosphate cleavage [15]. Each miRNA binds to specific mRNAs, inhibiting ribosomal access and subsequent translation to control gene expression [16] (see Figure 1). miRNA-mediated effects that lead to DICER1 syndrome may be due to loss-of-function in genes that normally contribute to the prevention of cancer (tumor suppressors), or alternatively, changes that lead to gain of function in genes that contribute to the onset of cancer in an active manner (oncogenes) [17]. Dysregulation of miRNA production is related to several tumor types [18]. Mutations in the *DICER1* gene are found in approximately 50–70% of pleuropulmonary blastoma patients [1].

Dicer plays additional diverse roles other than miRNA regulation [19]. Dicer plays a role in the processing of rRNA and, indirectly, ribosome biogenesis prior to export from the nucleus. In addition, Dicer plays a role in DNA processing and apoptosis [20]. Upon initiation of apoptosis, Dicer initiates the breakdown of chromosomal DNA, a key step in controlled cell death.

## 2. *DICER1* Germline Mutations

Research into the causal factors and mechanisms of DICER1 syndrome has been represented in the scientific literature since 2009. Generation and dissemination of new knowledge about DICER1 syndrome is increasing as demonstrated by the increasing number of publications each year over the past decade. Mutations that lead to DICER1 syndrome are reviewed here.

Pathogenic germline mutations of the *DICER1* gene linked to DICER1 syndrome are included in this review. Case studies provided detailed information regarding the germline mutation, patient(s), and physical manifestation. Mosaic *DICER1* mutations have also been associated with DICER1 syndrome [21], in addition to the inherited germline mutations. Cases represented in the PubMed database [22] were identified using keywords “DICER1 syndrome”, “DICER1 mutations”, and “Pleuropulmonary Blastoma Familial Tumor Susceptibility Syndrome”. A total of 244 articles were collected since the discovery of DICER1 syndrome, and 36 were selected that represented *DICER1* pathogenic germline mutations. Articles were excluded from this review if they did not address germline mutations in the *DICER1* gene, if they did not identify novel mutations, or if detailed documentation was absent from the report.

**Figure 2:**
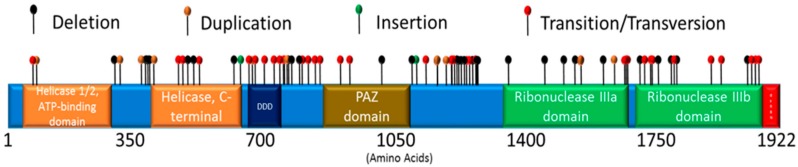
*DICER1* Pathogenic Germline Mutations. Mutations reported in *DICER1* include deletions, duplications, insertions, transitions, or transversions. The *DICER1* gene encodes 1922 amino acids, arranged into specific domains including the helicase 1/2, ATP-binding domain, the helicase, C-terminal domain, the Dicer dimerization domain (DDD), the PAZ domain (PAZ), the ribonuclease IIIa domain, and the ribonuclease IIIb domain. (see Tables 1–3).

Eighty-eight *DICER1* mutations are included in this review. While the majority of identified mutations are located within regions that encode one of Dicer’s seven defined domains (helicase domains, the Dicer dimerization domain (DDD), the Piwi/Argonaute, Zwille (PAZ) domain, the RNase III domains, and the double-stranded RNA-binding domain), some mutations were found to lie outside these domains (see Figure 2). Mutations, resulting protein changes, patient information, and background, clinical manifestation, and references are presented in Tables 1–3.

## 3. Manifestations of *DICER1* Gene Mutations

While the range of clinical symptoms associated with DICER1 syndrome is varied, some symptoms are prevalent among patients. These include multinodular goiter, pleuropulmonary blastoma, cystic nephroma, and ovarian Sertoli–Leydig Cell Tumor.

### 3.1. Multinodular Goiter (MNG)

Multinodular goiters, abnormal, cancerous growths of the thyroid gland, are associated with DICER1 syndrome and are a subsection of thyroid growths, which have been reported as a prevalent manifestation of DICER1 syndrome. Seventy-five percent of women and 17% of men with DICER1 syndrome were shown to harbor abnormal thyroid growths, multinodular goiters included, compared to the control population: 8% and 0% for women and men, respectively [23]. Specifically, a recent study indicated a correlation between truncating germline *DICER1* mutations and familial multinodular goiter, among other cancers [24]. Women are more likely to develop thyroid cancer than men, regardless of *DICER1* variant status [25]. Frequent malignant neoplasms of the endocrine system [26] such as thyroid cancers are linked to both environmental and genetic factors, with studies indicating similar links between multinodular goiters and both environmental and genetic factors [27]. *DICER1* mutations and subsequent global downregulation of miRNAs were found in multinodular goiter [28].

### 3.2. Pleuropulmonary Blastoma

Pleuropulmonary blastoma is a manifestation of DICER1 syndrome [29]. Primarily observed in children, it is a rare cancer that originates in the pleural cavity or the lungs [30]. Although rare, many cases of pleuropulmonary blastoma have been identified to be associated with DICER1 syndrome. First described in 1988 [31], cases are now documented in an international registry.

Four types of pleuropulmonary blastomas have been characterized. Type I is defined by cystic growths, has malignant potential, and may undergo malignant transformation in childhood. Type Ir is very similar to Type I, being defined by cystic growths, but contains no cancerous cells and is therefore not malignant. Type II consists of a hybrid of cystic and cancerous tumors, and type III solely consists of solid cancerous tumors. Types II and III have been associated with increased metastasis, primarily to the brain [32], and were found to be more aggressive than type I pleuropulmonary blastomas [33] (See Table 1).

### 3.3. Cystic Nephroma

Cystic nephromas have been reported in approximately 12% of children with pleuropulmonary blastomas or those with a family member with cystic nephroma [44,45]. The frequency of *DICER1* germline mutations in cystic nephroma patients was found to be 73.2% [46,47].

Cystic nephromas, multilocular cystic nephroma, and cystic renal hamartoma [48] are benign lesions in the kidney [49]. Common symptoms include hematuria, flank pain, and increased abdominal mass [50]. Enucleation of characteristic cysts can be associated with recurrence [51]. Treatment includes radical nephrectomy to prevent renal cell carcinoma [48].

A recent study of a patient with a cystic nephroma diagnosed as a multicystic left renal tumor was diagnosed with germline and somatic mutations in the *DICER1* gene [52]. Additionally, a nascent anaplastic sarcoma of the kidney (ASK) was reported within a cystic nephroma, associated with the presence of a germline *DICER1* mutation, or alternatively due to a somatic mutation [44,53].

### 3.4. Sertoli–Leydig Cell Tumor

Ovarian sex cord-stromal tumors have been associated with *DICER1* mutations [54,55], specifically, ovarian Sertoli–Leydig cell tumors. While rare among all ovarian neoplasms (<0.5%) [56], a recent study indicated that 57% of individuals with ovarian Sertoli–Leydig cell tumors also harbored *DICER1* germline mutations [46]. Another study confirmed this, indicating that more than 60% of ovarian Sertoli–Leydig cell tumors diagnosed harbored *DICER1* mutations within the RNase III domains [57]. A more recent study examined 34 Sertoli–Leydig cell tumors, with 88% containing one-or-more *DICER1* mutations [58].

Ovarian Sertoli–Leydig cell tumors are composed of several cell types, including Sertoli cells and Leydig cells [59]. They are responsible for an increase in testosterone production [60], and can lead to masculinization, voice deepening, and acne [61]. Interestingly, a recent study noted that the simultaneous occurrence of Sertoli–Leydig cell tumor and thyroid carcinoma is a reliable indicator of DICER1 syndrome [62] (See Table 2).

## 4. Additional Symptoms and Presentations Related to DICER1 Syndrome

### 4.1. Hodgkin Lymphoma

DICER1 syndrome includes novel symptoms which may facilitate early diagnosis. A rare form of Hodgkin lymphoma was diagnosed in an 11-year old boy with DICER1 syndrome in 2016 [41]. The patient had two *DICER1* mutations (c.5299delC and c.4616C>T), and several of his family members shared these mutations. Prior to this diagnosis, Hodgkin lymphoma had not been linked to DICER1 syndrome; additionally, this form of Hodgkin lymphoma is considered rare. Most Hodgkin and Reed–Sternberg cells arise from mature B cells, but a rare subset of cells arise from T cells. In this patient, the cells were found to be of the T-cell lineage, indicating a unique symptom. All affected family members developed at least one type of tumor with differing origins [5].

### 4.2. Pineoblastoma

Pineoblastoma may be associated with a *DICER1* mutation. Individuals with pineoblastomas were tested by de Kock and colleagues for the presence of *DICER1* mutations. They suggested that germline *DICER1* mutations make a clinically significant contribution to pineoblastoma; however, additional studies may confirm a causal relationship [69]. Additionally, the study of a single patient implicated a *DICER1* germline mutation in a pineoblastoma. The mutation was found to be heterozygous for germline but hemizygous in the tumor itself [70]. Further studies may determine the relationship between *DICER1* mutations and pineoblastomas (See Table 3).

### 4.3. Global Developmental Delay, Lung Cysts, Overgrowth, and Wilms Tumor (GLOW)

Documented by Klein and colleagues, symptoms include Global developmental delay, Lung cysts, Overgrowth, and Wilms tumor (GLOW). These symptoms were identified in patients with *DICER1* mutations in the RNase IIIb domain of Dicer [84]. Mutations were associated with Lung cysts and Wilms tumors, but also developmental delays and overgrowth, marked by large body size and mass. While both mutations reported were de novo missense mutations, the symptoms were similar to that of other patients diagnosed with germline mutations in similar loci within the *DICER1* gene. The current understanding of GLOW syndrome is limited, and more inquiry is required to determine how widespread GLOW syndrome is in relation to DICER1 syndrome.

### 4.4. Macrocephaly

A recently conducted study indicated that macrocephaly is associated with DICER1 syndrome [85]. Further studies are needed to confirm the link between macrocephaly and DICER1 syndrome, as this may help to identify individuals with DICER1 syndrome at an early stage.

## 5. Molecular Mechanisms of *DICER1* Mutations—The Two-Hit Hypothesis

The two-hit hypothesis, also known as the Knudson hypothesis, was originally suggested in 1953, and formally postulated by Knudson in 1971 [86,87]. The hypothesis suggests that, for abnormalities to arise, an individual requires two separate mutations in each allele, and that only one mutation in a single allele is not sufficient to induce the formation of tumors. Generally, a single mutation is inherited, which by itself is relatively harmless. However, a second mutation may act in tandem with the first to give rise to cancer. In a number of cases, the two-hit hypothesis describes the mechanism by which tumor suppressor gene deactivation occurs [88,89].

**Figure 3:**
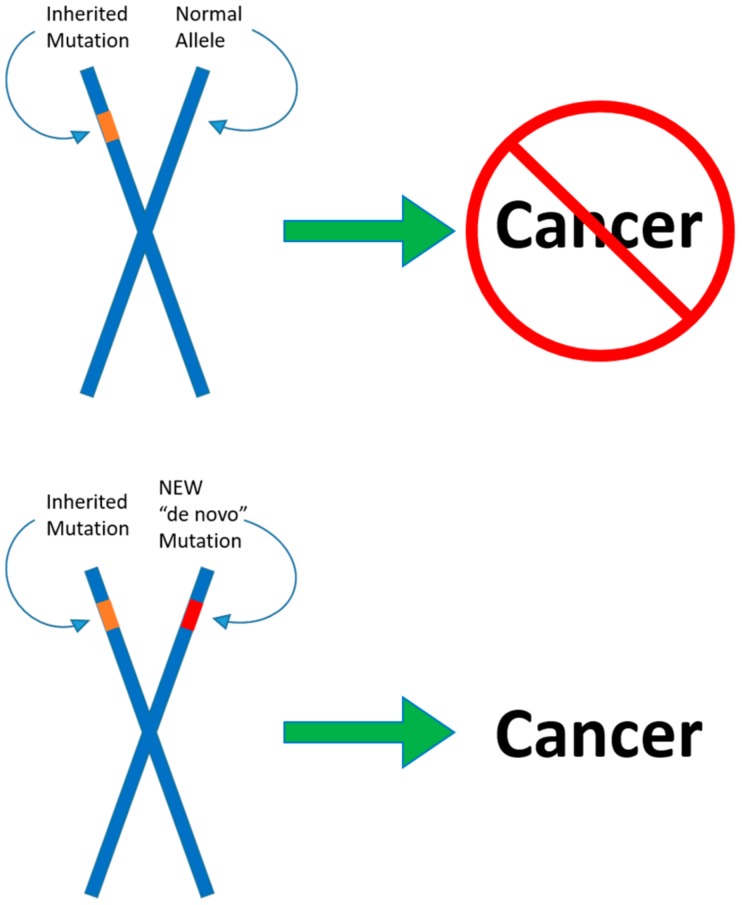
The two-hit hypothesis. One germline mutation in a *DICER1* allele predisposes the individual to an increased risk for benign and malignant tumors. A second somatic mutation in the other allele arising during tumorigenesis may lead to malignant rare cancers. While the first mutation by itself is overtly harmless, it only acts in tandem with the second to induce cancerous formation, according to the hypothesis.

DICER1 syndrome has been recognized as an autosomal-dominant disease, inherited and expressed in a haploinsufficient manner [1,5]. This proposed mechanism has been substantiated by a number of cases of individuals with DICER1 syndrome. Specifically, these cases involved individuals with only one apparent germline mutation, and symptoms characteristic of DICER1 syndrome. However, recent studies have indicated that patients with DICER1 syndrome have not only inherited mutations in one allele of the *DICER1* gene but also acquired a somatic mutation in the second allele of the *DICER1* gene. The two-hit hypothesis applies to *DICER1* mutations and the role of Dicer as a tumor suppressor gene (see Figure 3).

Second-hit, somatic mutations have been found in the RNase IIIb domain of the *DICER1* gene. A study involving three children with Wilms’ tumor suggested that the two-hit hypothesis applied to DICER1 syndrome, in the formation of Wilms’ tumor [90]. The patients were found to harbor germline *DICER1* mutations, and upon screening for somatic *DICER1* mutations, somatic mutations in the RNase IIIb domain on the second allele were found. Additionally, this finding highlights *DICER1* somatic gene mutations occurring in Wilms Tumor patients in the RNase IIIb domain. The regions of the gene encoding the RNase III domains are genetic hotspots for somatic mutations within the *DICER1* gene [91,92].

Biallelic *DICER1* mutations are common in pleuropulmonary blastomas, with the second mutation occurring within the RNase IIIb domain [93]. A study on 11 pleuropulmonary blastoma patients revealed that, out of 11 patients with *DICER1* gene mutations with sporadic pleuropulmonary blastomas, eight harbored biallelic *DICER1* gene mutations in which one of the mutations was within the RNase IIIb domain. 

A recent report of biallelic *DICER1* mutations in an ovarian fibrosarcoma from a 9-year-old patient demonstrated a germline single base insertion in the *DICER1* gene, causing a frameshift and premature stop codon as well as a second point mutation within the tumor that resulted in a substitution at amino acid position 1813 within the RNase IIIb domain (p.E1813G) [94].

Another case study involved a 14-month-old female patient diagnosed with pleuropulmonary blastoma and a previously removed cystic nephroma at 11 months [43]. Mutation analysis was conducted on available tissue and peripheral blood, revealing both alleles of the *DICER1* gene to be compromised. A missense heterozygous somatic mutation (c.5425G>A; p.G1809R) was detected in the DNA obtained from the cystic nephroma in addition to the germline truncating mutation detected from peripheral blood (c.5347C>T; p.Q1783*) within exon 24, which encodes the RNase IIIb domain. This germline mutation was confirmed in the patient’s mother and grandmother. In addition, the patient’s 21-year-old female cousin was found to also harbor the germline mutation and had previously been treated for embryonal rhabdomyosarcoma at age 14 and multimodal goiter at age 20. A unique missense heterozygous somatic mutation was detected in the embryonal rhabdomyosarcoma (c.5428G>C; p.D1810H) from a cousin of the patient. This case demonstrates that biallelic mutations in *DICER1* alleles, rather than haploinsufficiency, contribute to the mechanism of DICER1 syndrome.

Brenneman and colleagues [95] discussed the concept of biallelic mutations of the *DICER1* gene, with a focus on second, somatic, hot-spot mutations in the RNase IIIb domain. A cohort of individuals diagnosed with pleuropulmonary blastoma underwent analysis to determine the mutation status of *DICER1* alleles. Mutations within the RNase IIIb domain may represent hotspot mutations [96], and may be the rate-limiting step in the pathogenesis of DICER1 syndrome. Loss of function germline mutations in one allele of *DICER1* was found to be common among patients, and the RNase IIIb hotspot mutations were less common, but more frequently found within the tumor. RNase IIIb mutations may also predispose patients to additional mutations due to the role of Dicer in DNA replication and repair.

## 6. Future Directions

The breadth of knowledge regarding DICER1 syndrome continues to grow since its recent discovery, but promising treatments and management options need further investigation. As Dicer deficiency stems from *DICER1* mutations, experiments on Dicer in model animals have attempted to determine viable methods of upregulation of Dicer and related proteins to combat the effects of DICER1 syndrome. One such study conducted by Blandino and colleagues treated diabetic mice with metformin, reducing the incidence of cancer [97]. Mice treated with metformin showed reduced tumor growth and an upregulation of miRNAs and an increase in *DICER1* gene expression. A similar, recent experiment confirmed these results, as metformin was found to induce higher Dicer levels in mouse models and human patients by altering the localization of AUF1, a *DICER1* mRNA binding protein that down-regulates *DICER1*, leading to increased *DICER1* mRNA stability [98,99]. While the treatments described in these experiments may not be beneficial to patients with biallelic *DICER1* mutations, those with a single, functional DICER1 allele might benefit from metformin and compounds similar to it, as this single allele could hypothetically be targeted and upregulated to achieve increased Dicer protein production.

*DICER1* germline mutations have been identified as nonsense mutations, leading to stop codons within the coding sequence and truncated proteins or nonsense-mediated RNA degradation. While these mutations lead to cancerous and non-cancerous tumors, certain antibiotic treatments have been shown to promote the read-through of similar premature stop-codons, leading to restored transcription and translation of otherwise unreadable sequences [100]. One study demonstrated this novel approach by treating human cells containing known nonsense mutations with Ataluren, a pharmaceutical drug utilized in treating genetic disorders. Ataluren successfully promoted the read-through of all three nonsense codons within the mutated alleles [101]. A more recent study built upon these findings by modifying the fluorine number and position of Ataluren, which led to the increased read-through ability of stop codons in nonsense mutations [102]. While no research has been conducted on *DICER1* nonsense mutations utilizing drugs such as Ataluren, this drug and synonymous compounds may prove beneficial in treating patients with DICER1 syndrome stemming from nonsense mutations similar to those already tested. While treatments for pleuropulmonary blastoma and cystic nephroma have received wide attention from the scientific community, the root of these abnormal formations, *DICER1* germline mutations and acquired somatic mutations, require more study.

## 7. Conclusions

The mechanisms of DICER1 syndrome and its presentation in patients have become more fully understood over the past several years as more cases are diagnosed with novel mutations and presentations discovered and documented, extending the phenotypic range of this disorder. Successful treatments for DICER1 syndrome will require a combination of basic, translational, and clinical research. The outcome of the inaugural International DICER1 Symposium was the development of consensus testing and surveillance as well as treatment recommendations. Recommendations for genetic testing—including intronic sequencing [103]; whole exome sequencing [104]; or screening products such as the ThyroSeq, which is a DNA- and RNA-based next-generation sequencing assay that analyzes 112 genes for genetic alterations, including point mutations, insertions/deletions, gene fusions, and copy number alterations [105], prenatal management—and surveillance for DICER1-associated symptoms have been developed and described in recent publications [106,107,108].

## Figures and Tables

**Table 1:** Pathogenic germline mutations in the *DICER1* gene related to Pleuropulmonary Blastoma.

Mutation Type	Chromosomal Mutation	Protein Change	Clinical Manifestation	Reference
dup	c.1196_1197dupAG	p.Trp400Serfs*59	4-year old, pleuropulmonary blastoma.	Slade, 2011 [7]
tran	c.1376+1G>A	p.splice	13-year old female, peritoneal cysts of right & left round ligaments, nasal polyps, Sertoli–Leydig cell tumor. History: 5 years, type II pleuropulmonary blastoma, 8 years, thyroid nodules.	Schultz, 2016 [34]
tran	c.1507G>T	p.Glu503*	pleuropulmonary blastoma.	Hill, 2009 [1]
del	c.1684_1685delAT	p.Met562Valfs*11	pleuropulmonary blastoma.	Hill, 2009 [1]
del	c.1716delT	p.Phe572Leufs*15	0.8-year old, pleuropulmonary blastoma.	Slade, 2011 [7]
dup	c.1910dupA	p.Tyr637*	5-month old female, pleuropulmonary blastoma, and cervical embryonal rhabdomyosarcoma; pleuropulmonary blastoma & embryonal rhabdomyosarcoma.	Hill, 2009 [1]; Doros, 2012 [35]
tran	c.1966C>T	p.Arg656*	pleuropulmonary blastoma; 7-year old, pleuropulmonary blastoma.	Slade, 2011 [7] Hill, 2009 [1]
tran	c.2040+1G>T	p.splice	10-year female, nasal chondromesenchymal hamartoma. History: Pleuropulmonary blastoma.	Stewart, 2014 [36]
dup	c.2245_2248dupTACC	p.Pro750Leufs*12	pleuropulmonary blastoma.	Hill, 2009 [1]
tran	c.2247C>A	p.Tyr749*	pleuropulmonary blastoma.	Hill, 2009 [1]
del	c.2268_2271delTTTG	p.Cys756*	0.9-year old, pleuropulmonary blastoma.	Slade, 2011 [7]
tran	c.2379T>G	p.Tyr793*	11.5-years male, bilateral papillary thyroid carcinoma in follicular adenoma. History: 32 months, type II pleuropulmonary blastoma and cystic nephroma.	de Kock, 2014 [37]
dup	c.2392dupA	p.Thr798Asnfs*33	pleuropulmonary blastoma.	Hill, 2009 [1]
del	c.2399delG	p.Arg800fs*5	3.5-year old and 13-year old, Wilms’ tumor.	Palculict, 2016 [38]
tran	c.2830C>T	p.Arg944*	pleuropulmonary blastoma.	Hill, 2009 [1]
del	c.2863delA	p.Thr955fs	7-year old male, nasal chondromesenchymal hamartoma. History: pleuropulmonary blastoma.	Stewart, 2014 [36]
tran	c.3019C>T	p.Gln1007*	27-year old woman, nasal chondromesenchymal hamartoma and pleuropulmonary blastoma. History: multinodular goiter.	Stewart, 2014 [36]
del	c.3505delT	p.Ser1169Glnfs*23	3-year-old, Pleuropulmonary blastoma.	Slade, 2011 [7]
dup	c.3505dupT	p.Ser1169Phefs*8	7-year old female, thyroid goiter, multiple nodules on both lobes. History: 4.3 years, pleuropulmonary blastoma in left back musculature. 23 months, type II pleuropulmonary blastoma.	de Kock, 2014 [37]
tran	c.3540C>A	p.Tyr1180*	pleuropulmonary blastoma.	Hill, 2009 [1]
del	c.3583_3584delGA	N/A	6-year-old, intraocular medulloepithelioma. History: pleuropulmonary blastoma.	Slade, 2011 [7]
del	c.3665delT	p.Leu1222Tyrfs*17	4.2-year old, pleuropulmonary blastoma.	Slade, 2011 [7]
tran	c.3726C>A	p.Tyr1242*	4-year old, pleuropulmonary blastoma; 27-year old female, pleuropulmonary blastoma. History: 13 years, Sertoli–Leydig cell tumor and multinodular goiter, 21 years, nasal chondromesenchymal hamartoma.	Slade, 2011 [7]; Stewart, 2014 [36]
del	c.4309_4312delGACT	p.Asp1437Metfs*16	8-year old female, embryonal rhabdomyosarcoma. History: 4 years, pleuropulmonary blastoma; Median Age 34 months, female, cystic nephroma, pleuropulmonary blastoma.	Doros, 2012 [35]; Bahubeshi, 2010 [39]
del	c.4403_4406delCTCT	p.Ser1468Phefs*21	1.5-year old, pleuropulmonary blastoma.	Slade, 2011 [7]
del	c.4407_4410delTTCT	p.Leu1469fs	11-year old male, nasal chondromesenchymal hamartoma. History: pleuropulmonary blastoma.	Stewart, 2014 [36]
del	c.4555delG	p.Glu1519Lysfs*41	3-year old female, Polish, type II pleuropulmonary blastoma.	de Kock, 2013 [40]
tran	c.4616C>T	p.Thr1539Met	11-year-old male, Hodgkin lymphoma, pleuropulmonary blastoma Type I. History: thyroid cysts, syringomyelia.	Kuhlen, 2016 [41]
tran	c.4748T>G	p.Leu1583Arg	pleuropulmonary blastoma.	Hill, 2009 [1]
tran	c.5104C>T	p.Gln1702*	9-year old female, pleuropulmonary blastoma & ERMS.	Doros, 2012 [35]
del	c.5221_5232delAACAACACCATC	p.Asn1741_1744del	9-year old male, multinodular goiter, pleuropulmonary blastoma. History: 20 months, cystic nephroma.	Rath, 2014 [42]
del	c.5299delC	premature stop in exon 24	11-year-old male, Hodgkin lymphoma, pleuropulmonary blastoma Type I. History: thyroid cysts, syringomyelia.	Kuhlen, 2016 [41]
tran	c.5387C>T	p.Gln1783*	14-month old female, type I pleuropulmonary blastoma. History: cystic nephroma.	Fernandez-Martinez, 2017 [43]
tran	c.5465A>T	p.Asp1822Val	1.8-year-old, pleuropulmonary blastoma.	Slade, 2011 [7]
tran	c.5477C>A	p.Ser1826*	Median Age 34 months, female, cystic nephroma, pleuropulmonary blastoma.	Bahubeshi, 2010 [39]

del—deletion, dup—duplication, tran—transversion/transition.

**Table 2:** Pathogenic germline mutations in the *DICER1* gene related to Sertoli–Leydig Cell Tumor.

Mutation Type	Chromosomal Mutation	Protein Change	Clinical Manifestation	Reference
tran	c.325C>T	p.Gln109*	11-year old female, multinodular goiter. History: Sertoli–Leydig cell tumor.	Canfarotta M, 2016 [5]
del	c.876_879delAAAG	p.Arg293Ilefs*4	18-year old female, Sertoli–Leydig cell tumor. History: 16 years, multinodular goiter.	Rio Frio, 2011 [8]
tran	c.1376+1G>A	p.splice	13-year old female, peritoneal cysts of right & left round ligaments, nasal polyps, Sertoli–Leydig cell tumor. History: 5 years, type II pleuropulmonary blastoma, 8 years, thyroid nodules.	Schultz, 2016 [34]
del	c.1532_1533delAT	N/A	28-year old female, Sertoli–Leydig cell tumor. History: None | 16-year-old, Sertoli–Leydig cell tumor.	Oost, 2015 [63]
tran	c.2457C>G	p.Ile813_Tyr819del	32-year old female, Sertoli–Leydig cell tumor. History: 18 years, multinodular goiter.	Rio Frio, 2011 [8]
del/ins	c.3270-6_4051—1280delinsG	p.Tyr1091Ser*28	14-year old female, multinodular goiter. History: Sertoli–Leydig cell tumor, primitive neuroectodermal tumor.	Sabbaghian, 2013 [64]
tran	c.3540C>A	p.Tyr1180*	16-year old female, Sertoli–Leydig cell tumor. History: 14 years, bilateral multinodular goiter. 16-year old female, ovarian Sertoli–Leydig cell tumor, and lung lesion. History: 14 years, multinodular goiter.	de Kock, 2016 [65] Wu, 2014 [66]
tran	c.3647C>A	p.Ser1216*	13-year old female, Danish, multinodular goiter and Sertoli–Leydig cell tumor.	Rossing, 2014 [67]
tran	c.3649T>A	p.Tyr1217Asn	13-year old female, Danish, multinodular goiter and Sertoli–Leydig cell tumor.	Rossing, 2014 [67]
tran	c.3726C>A	p.Tyr1242*	27-year old female, pleuropulmonary blastoma. History: 13 years, Sertoli–Leydig cell tumor and multinodular goiter, 21 years, nasal chondromesenchymal hamartoma.	Stewart, 2014 [36]
del	c.4050+1delG	p.Val351Valfs*11	20-year old female, primitive neuroectodermal tumor & multinodular goiter. History: 9 years, Sertoli–Leydig cell tumor.	Foulkes, 2011 [68]
del	c.5018_5021delTCAA	p.Ile1673Thrfs*31	32-year old female, Sertoli–Leydig cell tumor. History: 18 years, multinodular goiter.	Rio Frio, 2011 [8]
del	c.5122_5128delGGAGATG	p.Gly1708Argfs*7	21-year old, Sertoli–Leydig cell tumor. History: 17 years, Sertoli–Leydig cell tumor.	Slade, 2011 [7]

del—deletion, dup—duplication, tran—transversion/transition.

**Table 3:** Pathogenic germline mutations in the *DICER1* gene related to cystic nephroma, pineoblastomas, Wilms’ tumor, multinodular goiter, medulloblastoma, rhabdomyosarcoma, pituitary blastoma, endometrial cancer, and seminoma.

Mutation Type	Chromosomal Mutation	Protein Change	Clinical Manifestation	Reference
dup	c.328_338dupGTGTCAGCTGT	p.Arg114Cysfs*18	3-year old, cystic nephroma.	Slade, 2011 [7]
dup	c.912_919dupAGACTGTC	p.Arg307Glnfs*8	4-year old male, Wilms’ tumor.	Foulkes, 2011 [68]
del	c.1128_1132delAGTAA	p.Lys376Asnfs*11	Pineoblastomas.	Sabbaghian, 2012 [70]
del	c.1153delC	p.Arg385Alafs*73	13-year old, Medulloblastoma/infratentorial primitive neuroectodermal tumor.	Slade, 2011 [7]
dup	c.1196_1197dupAG	p.Trp400Serfs*59	16-year old female, Ashkenazi Jewish/Anglo-Saxon, fibroadenoma of the breast. History: 6 years, ovarian embryonal rhabdomyosarcoma, 11 years, radiologic focal nodular liver hyperplasia, 12 years, cystic nephroma, 13 years, multinodular goiter.	de Kock, 2015 [71]
del	c.1284delGA	N/A	23-month old female, pituitary blastoma	de Kock, 2014 [72]
dup	c.1306dupT	p.Ser436Phefs*41	2-year old male, Wlims’ tumor.	Foulkes, 2011 [68]
tran	c.1525C>T	p.Arg509*	12-year old female, multinodular goiter. History: 6 years, dermoid cyst.	Darrat, 2013 [28]
tran	c.1966C>T	p.Arg656*	15-month old female, Pulmonary sequestration & cystic nephroma. 14-year old female, Belarusian-Serbian, hepatic focal nodular hyperplasia. History: Right Brain ventricle tumor (part teratoma, party embryonic carcinoma) at 8 months old. pilomatrixoma at 3 years, Renal cysts at 4 years, thyroid nodules at 10 years, basal cell carcinoma at 13 years.	Foulkes, 2011 [68] Mehraein, 2016 [73]
tran	c.2026C>T	N/A	17-year old female, pituitary blastoma.	de Kock, 2014 [72]
tran	c.2062C>T	p.Arg688*	8 year, a 9-month-old girl, anaplastic sarcoma of the kidney. History: pneumothorax, left upper lung cyst and left renal cyst at 10 months. cysts multiplied and increased in size over next few years.	Wu, 2016 [74]
tran	c.2117-1G>A	p.Gly706Aspsfs*8	10-year old female, multinodular goiter. History: 5 years, Wilms’ tumor.	Foulkes, 2011 [68]
tran	c.2247C>A	p.Tyr749*	6-week old male, embryonal rhabdomyosarcoma.	Doros, 2012 [35]
del	c.2399delG	p.Arg800fs*5	3.5-year old and 13-year old, Wilms’ tumor.	Palculict, 2016 [38]
tran	c.2407G>A	p.Gly803Arg	The average age of 44 months, Wilms’ tumor.	Palculict, 2016 [38]
del	c.2450delC	p.Pro817Leufs*15	7-month old female, Polish, multiseptated cystic mass in abdomen (early anaplastic sarcoma).	Wu, 2016 [52]
tran	c.2455T>C	p.Tyr819His	34 & 32-year old male family members, hepatocellular tumors.	Caruso, 2016 [75]
tran	c.2457C>G	p.Ile813_Tyr819del	53-year-old female, cERMS. History: multinodular goiter.	de Kock, 2015 [76]
tran	c.2516C>T	p.Ser839Phe	15-year old female, multinodular goiter.	Rio Frio, 2011 [8]
tran	c.2805-1G>T	p.Tyr936_Arg996del	The patient died at 20 years from alveolar rhabdomyosarcoma. History: multinodular goiter.	Rio Frio, 2011 [8]
del	c.3046delA	p.Ser1016Valfs*1065	12-month old female, pituitary blastoma.	Sahakitrungruang, 2014 [77]
tran	c.2379T>G	N/A	3-year old male, pituitary blastoma.	de Kock, 2014 [72]
del	c.3277_3280delAACT	N/A	7-year old female, pituitary blastoma.	de Kock, 2014 [72]
ins	c.3288_3289insTTTC	p.Gly1097Phefs*8	1.5-year old, cystic nephroma.	Slade, 2011 [7]
tran	c.3334A>G	p.Asn1112Asp	55-year old female, endometrial cancer.	Yang, 2015 [78]
dup	c.3405dupA	p.Gly1136Arg	12-year old female, renal cysts & focal nodular hyperplasia of the liver. History: 6 months, eRMS of the bladder and a cystic lesion in the lung at. 3 & 4.5 years, Ciliary body medulloepithelioma.	Fremerey, 2016 [79]
del	c.3535_3538delTCTT	p.Ser1179Thrfs*12	13-year old female, cervical sarcoma botryoides.	Tomiak, 2014 [80]
tran	c.3540C>G	p.Tyr1180	2-year old female, a multilocular cyst in left kidney, 2 cystic lesions in the lung, multicystic nephroma extended from left kidney.	Bardon-Cancho, 2016 [81]
del	c.3611_3616delACTACAinsT	p.Tyr1204Leufs*29	14-year old female, cERMS and thyroid goiter.	Foulkes, 2011 [68]
dup	c.3665dupT	p.Leu1222fs*13	30–39 year old female, soft tissue sarcoma	de Kock, 2017 [9]
del	c.3793delA	p.Thr1265Glnfs*37	6-year-old, ovarian sex cord stromal tumour.	Slade, 2011 [7]
del	c.3907_3908delCT	p.Leu1303Valfs*4	13-year old female, cervical embryonal rhabdomyosarcoma & two small lung cysts. History: 11 years, multinodular goiter.	Foulkes, 2011 [68]
del	c.4309_4312delGACT	N/A	Male, deceased 8 months post-surgery, pituitary blastoma.	de Kock, 2014 [72]
dup	c.4566_4579dupCTTTG	p.Val1524fs*38	14-month old female, neuroblastoma & cystic nephroma, multinodular goiter at age 7.	Saskin, 2017 [10]
tran	c.4740G>T	p.Gln1580His	32-year old, seminoma.	Slade, 2011 [7]
tran	c.5096-12G>A	N/A	10-year old female, undifferentiated sarcoma at ovary.	de Kock, 2017 [9]
tran	c.5125G>C(de novo)	N/A	21-month old male, pituitary blastoma.	de Kock, 2014 [72]
del	c.5221_5232delAACAACACCATC	p.Asn1741_1744del	9-year old male, multinodular goiter, pleuropulmonary blastoma. History: 20 months, cystic nephroma.	Rath, 2014 [42]
del/ins	c.5426_5442 del GGGATATTTTTGAGTCGinsCA	p.Gly1809_Ser1814delinsAla	15-year old female, thyroid follicular carcinoma. History: ASK for 12 years & multiple cystic-appearing thyroid nodules, no malignancy.	Yoshida, 2017 [82]
tran	c.5441C>T	p.Ser1814Leu	12.5-year-old female, ovarian tumor. History: 12 years, multinodular goiter.	Wu, 2016 [83]

del—deletion, dup—duplication, tran—transversion/transition.

## Abbreviations

AGO2	argonaute-2
AUF1	heterogeneous nuclear ribonucleoprotein D
DDD	Dicer dimerization domain
GLOW	Global developmental delay, Lung cysts, Overgrowth, and Wilms tumor
miRNAs	MicroRNA
PAZ	Piwi/Argonaute, Zwille domain
RISC	RNA-induced silencing complex
RLC	RNA-induced silencing complex (RISC) loading complex
RNase IIIb	ribonuclease IIIb
rRNA	ribosomal RNA
TARBP2	trans-activation responsive RNA binding protein 2
